# Gas signaling molecule hydrogen sulfide attenuates doxorubicin-induced dilated cardiomyopathy

**DOI:** 10.18632/oncotarget.20729

**Published:** 2017-09-08

**Authors:** Zongliang Yu, Wei Zhang, Mengyao Zhang, Mengchao Jin, Weiting Xu, Xiang Zhou

**Affiliations:** ^1^ Department of Cardiology, The First People's Hospital of Kunshan Affiliated to Jiangsu University, Kunshan, China; ^2^ Department of Cardiology, The Second Affiliated Hospital of Soochow University, Suzhou, China

**Keywords:** hydrogen sulfide, doxorubicin, dilated cardiomyopathy, oxidative stress, apoptosis

## Abstract

Increasing evidence has revealed that hydrogen sulfide (H_2_S) has beneficial effects in the treatment of various cardiovascular diseases. However, whether H_2_S can attenuate the development of dilated cardiomyopathy (DCM) remains unclear. In this study, we generated a rat model of DCM induced by doxorubicin and investigated the protective effects of H_2_S against DCM. Cardiac structure and function were analyzed by two-dimensional echocardiography. Oxidative stress was evaluated by measuring malondialdehyde, superoxide dismutase, glutathione peroxidase and reactive oxygen species. Cardiomyocyte apoptosis was assessed by flow cytometry following Annexin V/PI staining. Our results showed that exogenous administration of H_2_S could improve left ventricular structure and function in DCM rats. H_2_S was found to suppress doxorubicin-induced oxidative stress by activating the Nrf2 pathway and upregulating the expression of antioxidant proteins NQO1 and GCLM. Moreover, H_2_S was also found to inhibit doxorubicin-induced cardiomyocyte apoptosis by activating the PI3K/Akt signaling pathway. In conclusion, our study demonstrates that H_2_S protects against doxorubicin-induced DCM via attenuation of oxidative stress and apoptosis.

## INTRODUCTION

Dilated cardiomyopathy (DCM), a severe disorder characterized by ventricular enlargement and systolic dysfunction, is a crucial cause of sudden cardiac death and congestive heart failure [1]. Numerous factors have been identified associated with the development of DCM, such as long term use of doxorubicin for cancer chemotherapy. It has been well documented that doxorubicin can increase oxidative stress, reduce ATP yield, alter gene expression and inducecardiomyocyte apoptosis, which are involved in the pathogenesis of DCM [2].

Hydrogen sulfide (H_2_S) is emerging as a new gaseous signaling molecule which exerts multifactorial effects on various intracellular signaling pathways [3, 4]. In the cardiovascular system, H_2_S is generated in the myocardial cells and blood vessels from L-cysteine by the enzyme cystathionine γ-lyase. In recent years, there is growing evidence that H_2_S plays critical roles in the regulation of cardiovascular physiology and pathology [5, 6]. H_2_S was found to reduce myocardial ischemia-reperfusion injury by preserving mitochondrial function and to attenuate ischemia-induced heart failure by decreasing oxidative stress [7, 8]. In addition, H_2_S could inhibit cardiac hypertrophy and myocardial fibrosis and improve left ventricular function in diabetic rats [9].

In the present study, we established a rat model of DCM induced by doxorubicin to investigate whether H_2_S has the protective effect against DCM. Our findings indicated that H_2_S could attenuate the progression of DCM by suppressing oxidative stress and cellular apoptosis.

## RESULTS

**Figure 1:**
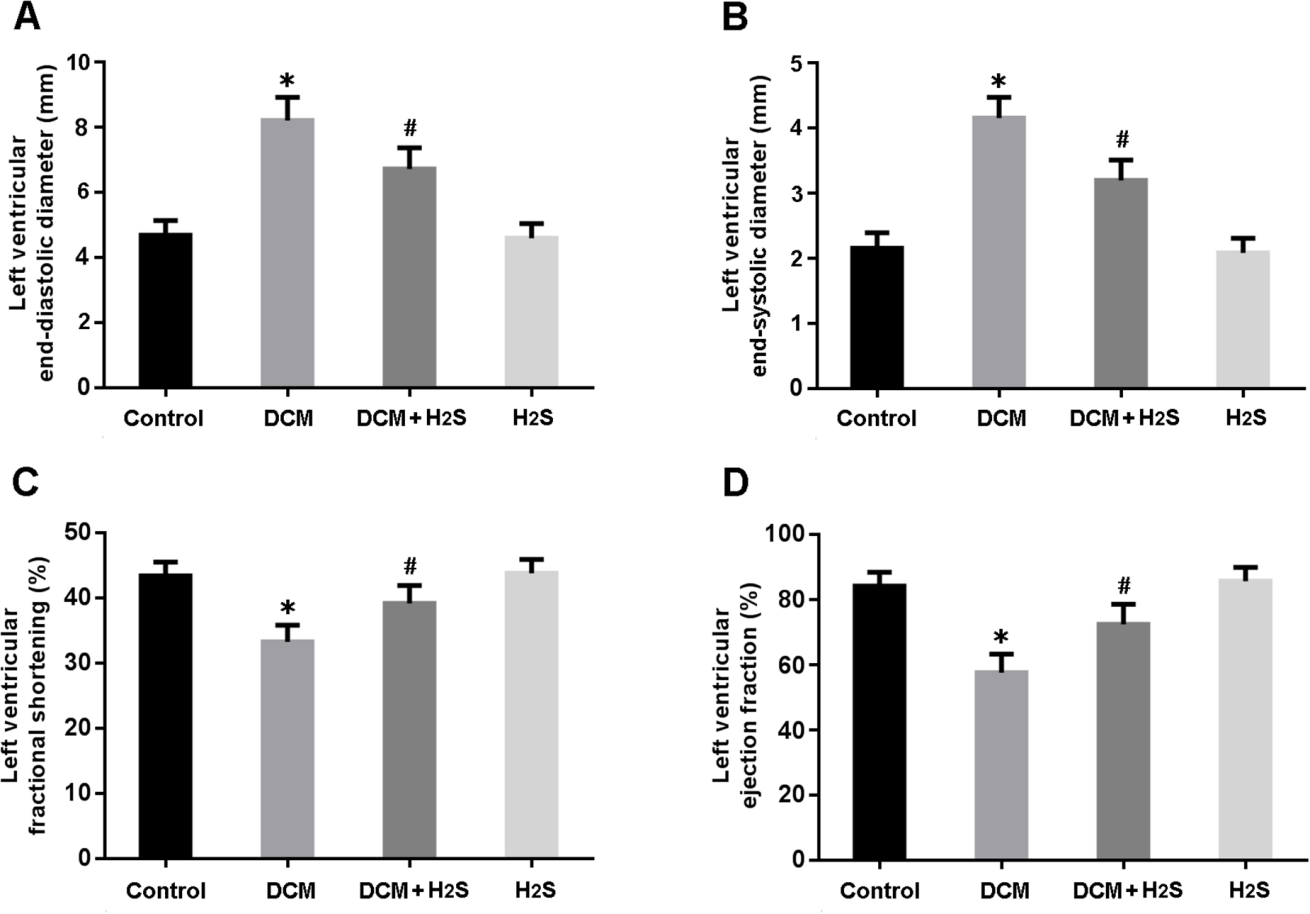
Echocardiographic evaluation of cardiac structure and function **(A)** left ventricular end-diastolic diameter; **(B)** left ventricular end-systolic diameter; **(C)** left ventricular fractional shortening; **(D)** left ventricular ejection fraction. ^*^ P < 0.05, vs. control; ^#^ P < 0.05, vs. DCM (n = 5).

Cardiac structure and function were analyzed by echocardiography. LVEDD and LVESD were significantly increased in the DCM group and decreased in the DCM + H_2_S group (Figure 1A, 1B). Moreover, LVFS and LVEF were found to be markedly lower in the DCM rats, whereas exogenous administration of H_2_S could improve left ventricular systolic function (Figure 1C, 1D).

**Figure 2:**
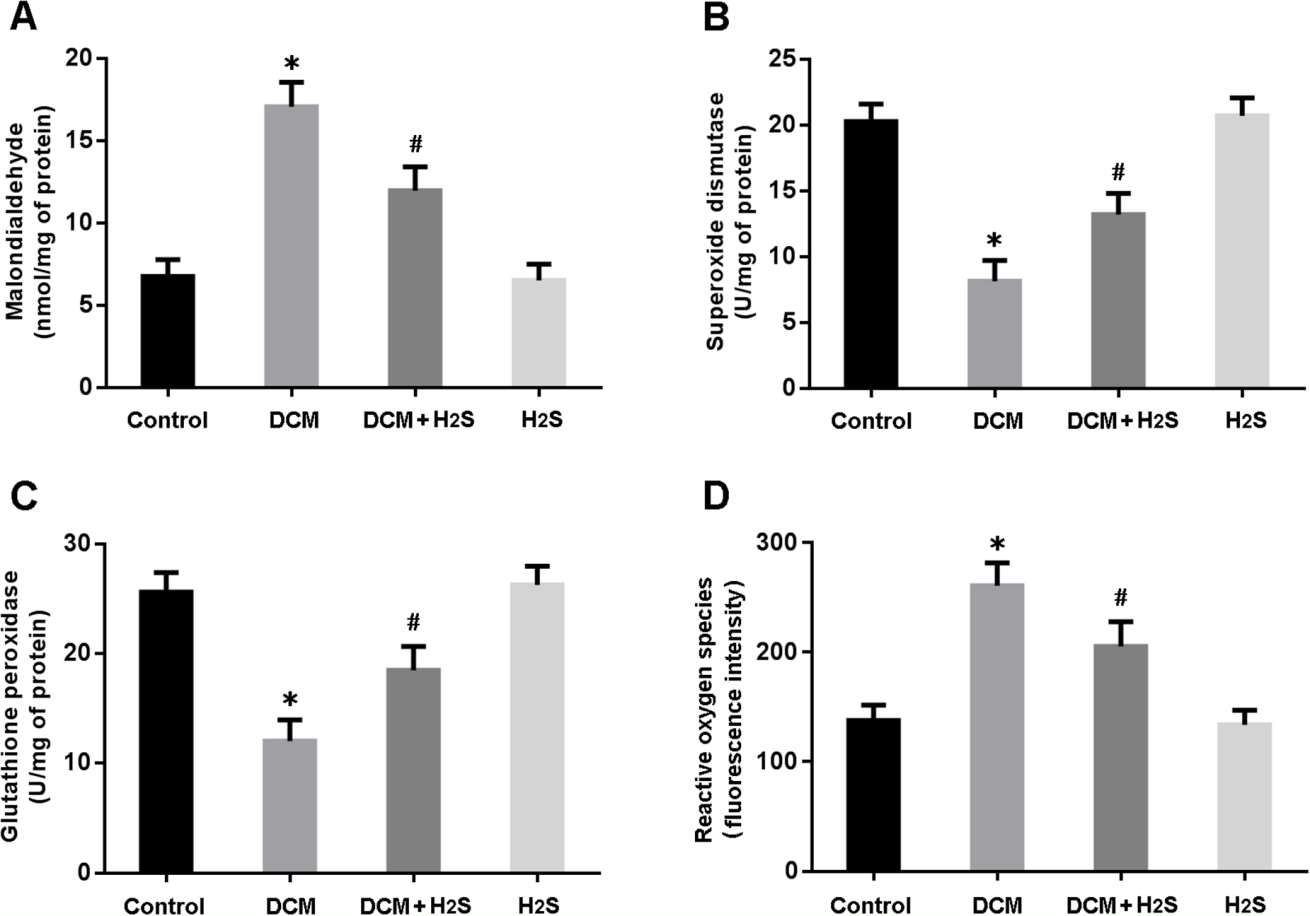
Measurements of oxidative stress in myocardium **(A)** malondialdehyde; **(B)** superoxide dismutase; **(C)** glutathione peroxidase; **(D)** reactive oxygen species. ^*^ P < 0.05, vs. control; ^#^P < 0.05, vs. DCM (n = 5).

Oxidative stress was assessed by measuring MDA levels, SOD and GSH-Px activities and ROS production in the myocardium. There were significant increase in MDA levels and decrease in SOD and GSH-Px activities in DCM rats, while NaHS administration could reduce MDA levels and increase SOD and GSH-Px activities (Figure 2A-2C). Moreover, the ROS generation in cardiac tissue was elevated in the DCM group and decreased in the DCM + H_2_S group (Figure 2D).

**Figure 3:**
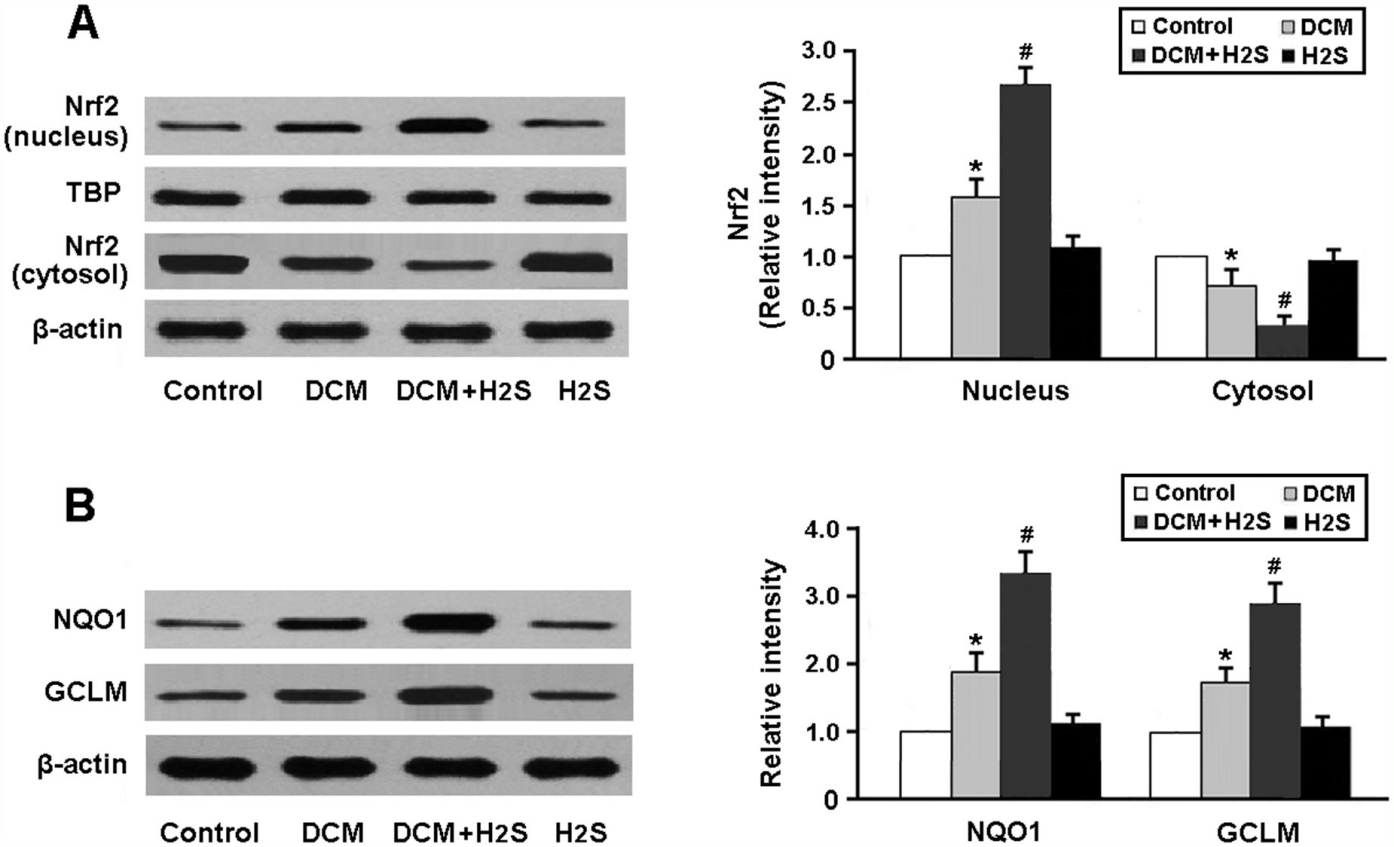
Representative immunoblots and densitometric analysis of Nrf2 in the nucleus and cytosol **(A)** and its downstream targets NQO1 and GCLM **(B)**. ^*^ P < 0.05, vs. control; ^#^ P < 0.05, vs. DCM (n = 5).

The Nrf2 pathway was detected by Western blotting. As shown in Figure 3A, the nuclear expression of Nrf2 protein was significantly increased in the myocardium of DCM rats following treatment with NaHS. In addition, the protein expression of downstream targets of Nrf2, NQO1 and GCLM, was markedly upregulated in the DCM + H_2_S group (Figure 3B).

**Figure 4:**
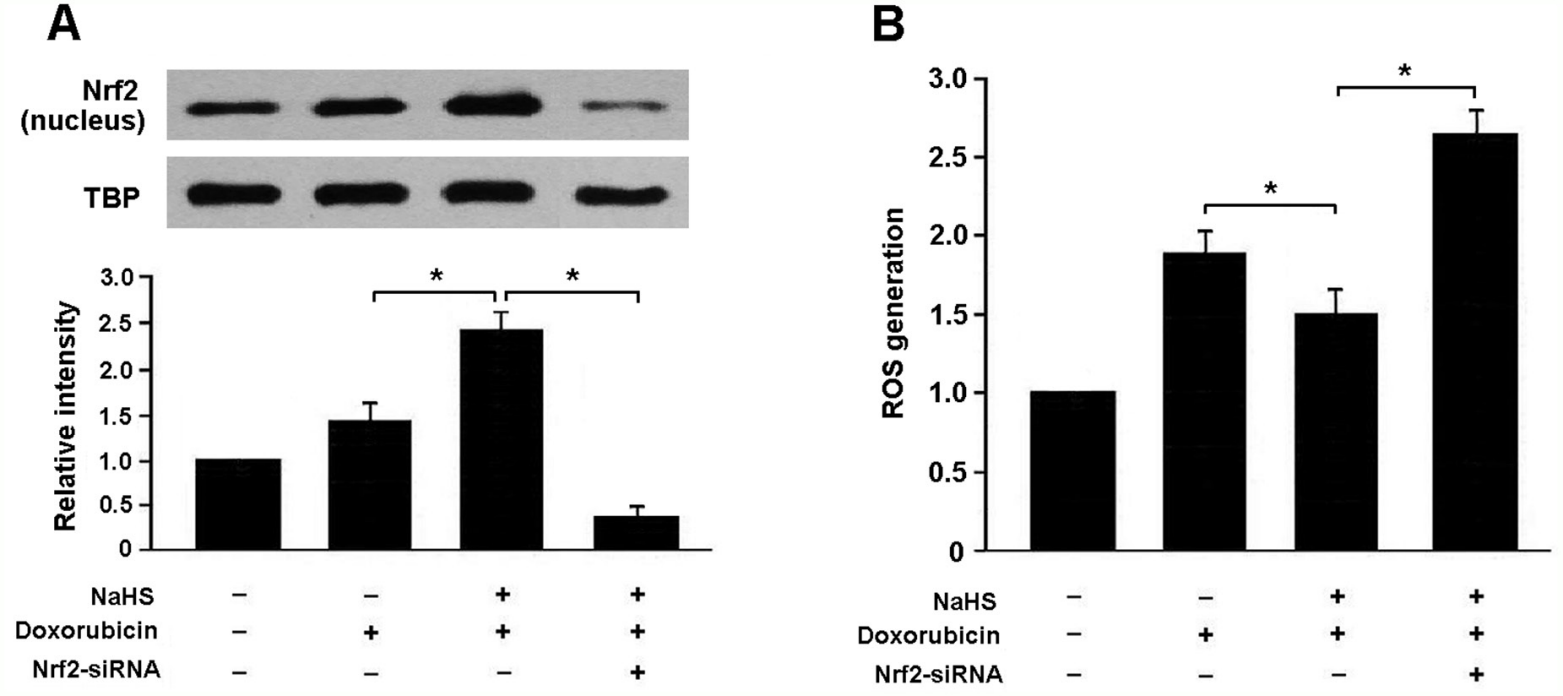
**(A)** Representative immunoblots and densitometric analysis of Nrf2 in neonatal rat cardiomyocytes; **(B)** Reactive oxygen species (ROS) generation in cardiomyocytes with different treatment; NaHS (100 μmol/L) and doxorubicin (1 μmol/L). ^*^ P < 0.05 (n = 3 independent experiments).

To verify whether H_2_S attenuates doxorubicin-induced oxidative stress in Nrf2-dependent manner, we transfected myocardial cells with Nrf2 siRNA and then subjected them to doxorubicin and NaHS. Our results indicated that Nrf2 siRNA-transfected cells that exposed to doxorubicin and NaHS exhibited decreased expression of Nrf2 and increased generation of ROS compared to cells without Nrf2 siRNA transfection (Figure 4A, 4B).

**Figure 5:**
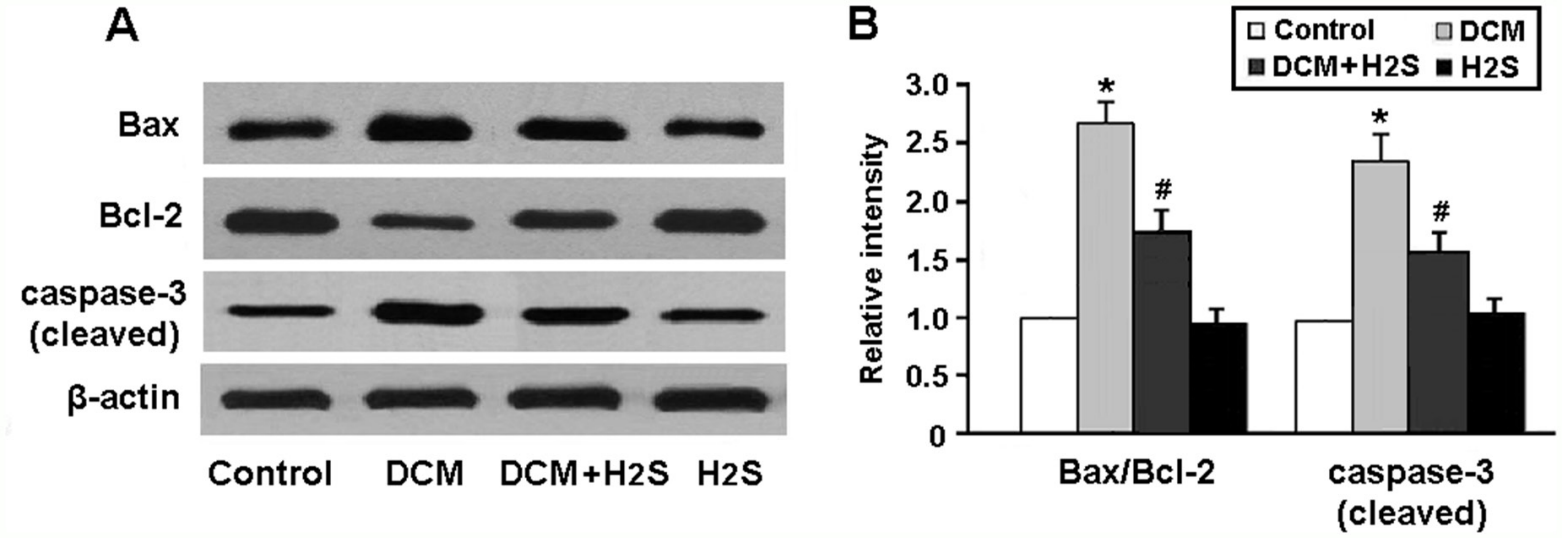
**(A, B)** Representative immunoblots and densitometric analysis of apoptotic regulatory proteins Bax, Bcl-2 and caspase-3. ^*^ P < 0.05, vs. control; ^#^ P < 0.05, vs. DCM (n = 5).

Cardiomyocyte apoptosis was determined by detecting the expression of apoptotic regulatory proteins in rats. Our results showed that the Bax/Bcl-2 ratio and cleaved caspase-3 expression were significantly increased in the myocardium of DCM rats and reduced after treatment with NaHS (Figure 5A, 5B).

**Figure 6:**
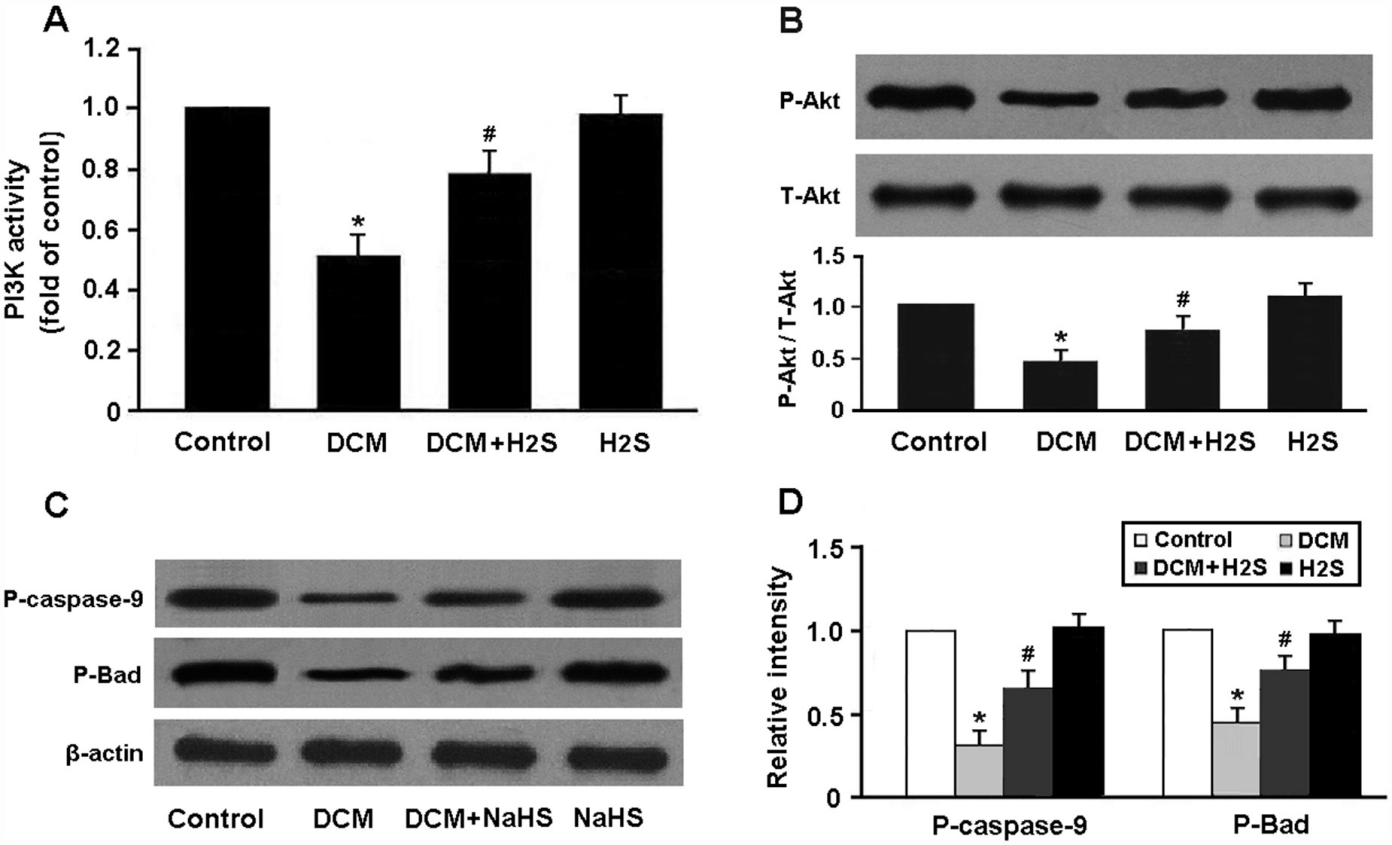
**(A)** PI3K activity in myocardium was measured by ELISA; **(B-D)** Western blot analysis of Akt phosphorylation and its downstream targets phospho-caspase-9 and phospho-Bad. ^*^ P < 0.05, vs. control; ^#^ P < 0.05, vs. DCM (n = 5).

PI3K activity was found to be remarkably decreased in the DCM group and elevated in the DCM + H_2_S group (Figure 6A). In addition, the expression of phospho-Akt and its downstream target proteins, phospho-caspase-9 and phospho-Bad, was downregulated in the DCM rats and upregulated following administration of NaHS (Figure 6B-6D).

**Figure 7:**
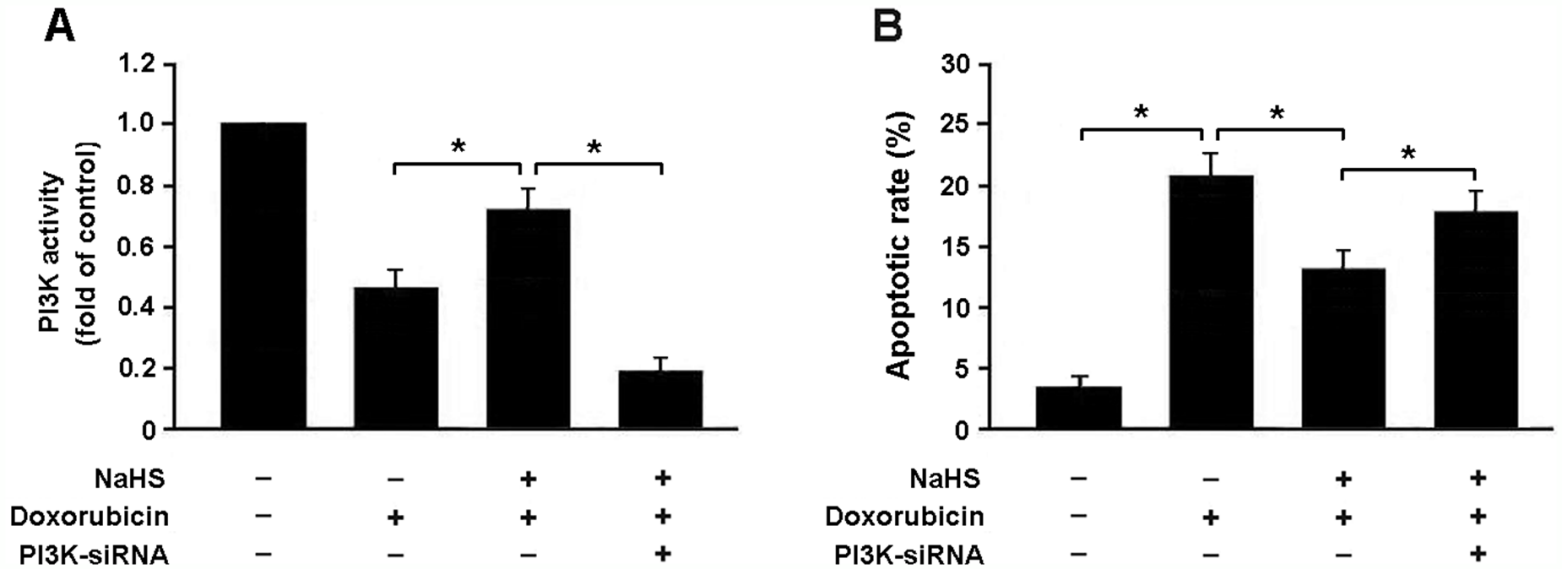
**(A)** PI3K activity in neonatal rat cardiomyocytes was detected by ELISA; **(B)** Apoptotic rate was determined by flow cytometry in cardiomyocytes with different treatment; NaHS (100 μmol/L) and doxorubicin (1 μmol/L). ^*^ P < 0.05 (n = 3 independent experiments).

To confirm whether H_2_S attenuates doxorubicin-induced apoptosis by activating PI3K/Akt pathway, we transfected cardiomyocytes with PI3K siRNA and then subjected them to doxorubicin and NaHS. Our results indicated that PI3K siRNA-transfected cells that exposed to doxorubicin and NaHS exhibited reduced expression of PI3K and increased apoptotic rate compared to cells without Nrf2 siRNA transfection (Figure 7A, 7B).

## DISCUSSION

In the present study, we established a rat model of DCM induced by doxorubicin and found that exogenous administration of NaHS (a donor of H_2_S) could improve cardiac structure and function in DCM rats. Moreover, our findings also revealed that H_2_S could attenuate doxorubicin-induced DCM by inhibiting oxidative stress and cardiomyocyte apoptosis.

There is growing evidence that oxidative stress plays important roles in doxorubicin-induced subcellular remodeling, Ca^2+^-handling abnormalities and mitochondrial dysfunction, eventually leading to cardiac dysfunction, with subsequent congestive heart failure [10, 11]. ROS generation associates with intracellular Ca^2+^ accumulation and leads to the collapse of mitochondrial membrane potential. The subsequent release of cytochrome c from mitochondria to cytosol is correlated with doxorubicin-mediated apoptosis [12]. Nrf2 is a member of the NF-E2 family of transcription factors and can modulate the expression of several antioxidative enzymes [13]. In response to oxidative stress, Nrf2 is free from Keap1 and translocates into the nucleus to bind to ARE in the promoters of genes encoding antioxidant enzymes [14]. In this study, H_2_S was found to enhance the binding activity of Nrf2-ARE and upregulate the expression of antioxidant enzymes NQO1 and GCLM, which consequently increased the resistance to oxidative stress in DCM.

It is generally accepted that cardiomyocyte apoptosis is involved in the pathogenesis of doxorubicin-induced DCM [15]. Doxorubicin can induce apoptosis by activating mitochondria apoptosis pathway. Transducing cardiomyocytes with Hsp10 or Hsp60 reduced the occurrence of apoptosis in doxorubicin-treated myocardial cells [16]. Cardiomyocyte-specific deletion of Top2b protected against doxorubicin-induced DNA double-strand breaks and transcriptome changes and improved ventricular function [17]. PI3K/Akt signaling pathway plays critical roles in the regulation of cell proliferation and survival by phosphorylating various substrates, including Ikappa B kinase, Bad, caspase-9 and forkhead transcription factors [18]. In this study, PI3K/Akt signaling was found to be inhibited in the myocardium of DCM rats, which might be an important molecular mechanism responsible for cardiomyocyte apoptosis. Moreover, H_2_S was found to decrease doxorubicin-induced apoptosis by activating PI3K/Akt pathway.

In summary, our study demonstrates that H_2_S alleviates the development of DCM via attenuation of oxidative stress and apoptosis. H_2_S may reduce doxorubicin-induced oxidative stress by activating Nrf2 signaling and may exert antiapoptotic effects in DCM by activating PI3K/Akt pathway.

## MATERIALS AND METHODS

### Animal model and grouping

All experiments were approved by the Animal Ethics Committee of Soochow University. DCM was induced by doxorubicin as previously described [19]. Briefly, male Sprague-Dawley rats weighing 200-250g were intraperitoneally administered with doxorubicin hydrochloride in six equal injections (each containing 2.5 mg/kg) over a period of 2 weeks for a total cumulative dose of 15 mg/kg. Two weeks after cessation of doxorubicin injection, rats were examined by transthoracic echocardiography to confirm DCM. The rats were then divided into 4 groups: control group, DCM group, DCM + H_2_S group (DCM rats were administrated with NaHS solution at a dose of 15 μmol/kg/d), and H_2_S group (normal rats were administered with H_2_S donor NaHS). After 12 weeks, animals were sacrificed by cervical dislocation, and the hearts were harvested for analysis.

### Cardiomyocyte culture

The hearts were surgically removed from 1-3 days old Sprague-Dawley rats, washed and minced in D-Hanks solution. Cardiac tissues were then dispersed in a series of incubations at 37°C in D-Hanks solution containing 1.2 mg/ml pancreatin and 0.14 mg/ml collagenase (Gibco, USA). After centrifugation, the cells were suspended in Dulbecco's modified Eagle's medium (Gibco, USA) containing 20% calf serum, 100 U/ml penicillin and 100 μg/ml streptomycin. The dissociated cells were preplated at 37 °C for 1 h to separate cardiomyocytes by adherence of cardiac fibroblasts. Thereafter, cells were collected and diluted to 1×10^6^ cells/ml and plated onto 1% gelatin-coated culture dishes.

### Echocardiographic study

Left parasternal and left apical echocardiographic images were obtained using a Philips ultrasound system. Left ventricular end-diastolic diameter (LVEDD) and left ventricular end-systolic diameter (LVESD) were measured from the parasternal long-axis view. Left ventricular fractional shortening (LVFS) and left ventricular ejection fraction (LVEF) were determined to evaluate left ventricular systolic function. All measurements were performed by the same observer based on the average of three consecutive cardiac cycles.

### Measurement of oxidative stress

Oxidative stress was assessed by detecting malondialdehyde (MDA) levels, superoxide dismutase (SOD) and glutathione peroxidase (GSH-Px) activities, and reactive oxygen species (ROS) production in the myocardium according to the instructions of detection kits (Jiancheng Biotech, China).

### Annexin V-FITC/PI staining

Cellular apoptosis was determined using the Annexin V-FITC apoptosis detection kit (BD Pharmingen, USA) according to the manufacturer's instructions. Cardiomyocytes were stained with Annexin V-FITC and propidium iodide (PI) and then subjected to flow cytometry analysis.

### PI3K activity assay

PI3K activity was determined using an enzyme-linked immunosorbent assay (ELISA) kit purchased from Echelon Biosciences (Salt Lake City, UT). In this method, PI3K activity was assessed by detecting the conversion of PI(4,5)P2 into PI(3,4,5)P3.

### Western blotting

Proteins from myocardial tissue and cardiomyocytes were extracted in RIPA buffer containing phosphatase and protease inhibitor cocktail (Thermo Scientific). The protein extracts were subjected to centrifugation at 12 000 g for 15 min and loaded onto sodium dodecyl sulfate-polyacrylamide gels and then transferred to nitrocellulose membranes. After blocking with 5% nonfat milk in TBS containing 0.1% Tween-20, the membranes were incubated with primary antibodies overnight at 4 °C, followed by incubation with HRP-conjugated secondary antibodies. The antibodies were purchased from Cell Signaling Technology and were used at manufacturer's recommended dilutions. Finally, the signal was detected using the enhanced chemiluminescence kit (Amersham Biosciences, Piscataway, NJ).

### Statistical analysis

The data are presented as mean ± SD, and the differences between groups were compared using one-way ANOVA with SPSS version 18.0. The Scheffé post hoc test was used for multiple comparisons if the ANOVA was significant. A value of P < 0.05 was considered statistically significant.
